# DASH diet, Reduced Rank Regression Dietary Patterns and relations with kidney function in the CHRIS general population study

**DOI:** 10.1186/s12882-026-04896-z

**Published:** 2026-04-20

**Authors:** Giulia Barbieri, Vanessa Garcia-Larsen, Rebecca Lundin, Ryosuke Fujii, Pietro Manuel Ferraro, Giovanni Gambaro, Roberto Melotti, Martin Gögele, Lucia Cazzoletti, Peter P. Pramstaller, Maria Elisabetta Zanolin, Cristian Pattaro, Essi Hantikainen

**Affiliations:** 1 Institute for Biomedicine, Eurac Research, Via Volta, 21, 39100 Bolzano, Italy; 2 Unit of Epidemiology and Medical Statistics, Department of Diagnostics and Public Health, University of Verona, Verona, Italy; 3 Department of International Health, The Johns Hopkins Bloomberg School of Public Health, Baltimore, MD USA; 4 Department of Preventive Medical Sciences, Fujita Health University School of Medical Sciences, Toyoake, Japan; 5 Division of Nephrology, Department of Medicine, University of Verona, Verona, Italy

**Keywords:** CHRIS study, Dietary Patterns, Chronic kidney disease, Reduced Rank Regression, Kidney function, Food frequency questionnaire, Diet, DASH diet

## Abstract

**Background:**

Chronic kidney disease (CKD) is a growing public health concern, closely linked to aging and chronic conditions such as diabetes, cardiovascular disease, and hypertension. Diet is a modifiable risk factor for kidney function, but evidence on how specific dietary patterns (DPs) relate to kidney health in healthy populations remains limited. To address this, we evaluated associations between DPs and kidney function, considering sex and menstrual status. DPs were derived using both an a priori approach (DASH) and a hybrid method (RRR), capturing established dietary recommendations as well as population-specific behaviours relevant to kidney health.

**Methods:**

We analysed cross-sectional data from 6133 healthy adult participants of the Cooperative Health Research In South Tyrol (CHRIS) study. Participants self-reporting previous diagnoses of any kidney disease, hypertension, or diabetes were excluded. Using self-reported food frequency questionnaire data, we derived the DASH-score and two RRR-based sex-specific DP-scores based on nine cardio-renal-metabolic parameters. We applied sex-stratified linear and non-linear models to examine associations with creatinine-based eGFR, including interaction and stratified analyses by menstrual status in females.

**Results:**

In males, a DP reflecting high intake of cereals, whole grains, sugar, fruits, and legumes, and low intake of beer, red and processed meat was associated with higher eGFR levels (β = 0.75, *p* = 0.0001). Similarly, higher adherence to the DASH diet was also positively associated with eGFR (β = 0.64, *p* = 0.0029). In females, the associations varied by menstrual status. Among those with ceased menstruation, a DP reflecting low intake of meat, spirits, and refined grains, and high intake of whole grains and dairy products was associated with higher eGFR (β = 1.11, *p* = 0.0049). In females still experiencing regular menstruation, a DP reflecting high intake of beef, nuts, beer, and legumes, and low intake of refined grains was associated with lower eGFR (β = −0.43, *p* = 0.0326). No association was observed with the DASH score in females.

**Conclusions:**

The DASH-style diet is associated with better kidney function in males, but not in females. Identifying sex-specific kidney function-oriented DPs using RRR provides new insights into the diet-eGFR relationship, suggesting potential effect modification by menstrual status.

**Supplementary information:**

The online version contains supplementary material available at 10.1186/s12882-026-04896-z.

## Introduction

Already affecting approximately 10% of the global adult population, chronic kidney disease (CKD) has become a major public health concern [1]. The increasing prevalence of chronic conditions like diabetes, cardiovascular disease (CVD), and hypertension, alongside population aging, are the most recognized factors contributing to the growing CKD burden [2]. Moreover, obesity may lead to CKD either directly through metabolic alterations or indirectly through the development of diabetes and hypertension [3]. Prevention is thus fundamental to reduce exposure to avoidable risk factors [4].

Biological sex and gender are key modifiers of kidney physiology and disease progression [5], as well as recognition, monitoring and management of CKD [6]. However, these dimensions remain underexplored in both clinical research and practice, despite growing evidence of their influence [7]. Sex-specific differences in dietary habits, renal structure, hemodynamics, and hormonal regulation could influence how individuals respond to dietary exposures and disease risk [8]. In women, the transition through menopause marks a significant hormonal shift, particularly the decline in estradiol, which has been associated with accelerated kidney function decline [9]. Additionally, ferroptosis, a regulated form of cell death driven by iron accumulation and lipid peroxidation, has been implicated in both acute and chronic kidney injury [10, 11]. Given that iron metabolism and oxidative stress responses vary by sex and hormonal status, stratifying analyses by sex and menopausal status is essential to uncover biologically meaningful interactions and to inform more precise dietary and therapeutic strategies.

CVD and CKD share several interconnected biological pathways, including chronic low‑grade inflammation, oxidative stress, and endothelial dysfunction, which contribute to their frequent co‑occurrence. For this reason, lifestyle recommendations often coincide, and similar dietary recommendations are often suggested [12]. The Dietary Approach to Stop Hypertension (DASH) Diet, characterized by higher intake of plant-based foods, has been specifically designed to prevent and manage high blood pressure, and has further been recommended for non-dialytic CKD patients to improve kidney health [13]. Moreover, general population studies suggest a protective association between the DASH diet and risk of CKD onset and progression [14, 15]. Beyond its antihypertensive effects, the DASH diet may also support kidney health through additional pathways, including lowering dietary acid load via higher fruit and vegetable intake [16], promoting a more favourable mineral balance, and reducing sodium intake, which may indirectly reduce intraglomerular pressure [17]. Nevertheless, the pathophysiological role of diet and its components in kidney function is not yet fully ascertained.

Other methodological approaches to estimate dietary patterns, such as Reduced Rank Regression (RRR), have emerged as useful as they implement prior knowledge of the diet-disease pathway and allow identification of population-specific dietary patterns (DPs) [18–20]. This is particularly relevant in the context of CKD prevention, where a-priori defined dietary indices have not yet been established and validated.

To date, only two studies have applied RRR in relation to kidney health using biomarkers as mediators. Cai et al. applied RRR to investigate associations between eGFR-based DPs and CKD outcomes in a CKD-free population-based cohort [21]. Kurniawan et al. investigated RRR-based DPs in association with severity of impaired kidney function in a population-based study among participants with CKD [22]. In this investigation, we derived RRR-based DPs using nine cardio-renal-metabolic biomarkers, which are established risk factors for reduced eGFR, as mediators. To gain a more complementary and comprehensive understanding of how diet relates to eGFR, we assessed the associations of both the DASH diet score and the RRR-derived DPs with eGFR. Analyses were conducted using cross-sectional data from a large, generally healthy population sample. Additionally, we explored potential effect modification by sex and menstrual status.

## Methods

### Study design

Analyses were conducted within the Cooperative Health Research in South Tyrol (CHRIS) study, a population-based study that recruited 13,393 adults from the Vinschgau/Val Venosta district (South Tyrol, Italy) between 2011 and 2018, as extensively described elsewhere [23, 24]. Participants were invited to the study centre in the early morning following overnight fasting. They underwent blood drawing, urine collection, anthropometric analysis, blood pressure measurements, clinical examinations, and standardized self- and interviewer-administered questionnaires about medical history and lifestyle. Further details about the CHRIS study design and the types of information collected, including the specific questionnaires, are provided in the CHRIS study protocol paper [23]. The validated Global Allergy and Asthma Network of Excellence (GA^2^LEN) food frequency questionnaire (FFQ) [25], was introduced in 2014 and filled out by 8842 participants.

We excluded participants reporting a diagnosis of any kidney disease [26], hypertension, or diabetes, according to the questionnaire interview or the provision of corresponding medications (Supplementary Table S1). We further excluded 5 participants with >80% missing laboratory biomarker data, 34 participants with >20% missing FFQ items, and 86 out of the 0.5^th^-99.5^th^ percentile range of the total energy intake (TEI)/basal metabolic rate ratio (see flowchart in Supplementary Figure S1). Residual missing values in the FFQ were assumed to indicate non-consumption and imputed as zeros [27]. Missingness in all other variables (0.9% globally) was handled with Multiple Imputations by Chained Equations (MICE) using the ‘mice’ R package v3.14.0 (https://cran.r-project.org/web/packages/mice/index.html). Sample characteristics remained stable between the imputed and unimputed datasets. Eventually, 6133 individuals were available for analysis.

### Outcome

The outcome of our analyses was eGFR, estimated from serum creatinine using the 2021 CKD-EPI equation, using the R package ‘nephro’ v1.3 (https://cran.r-project.org/web/packages/nephro/index.html).

### Assessment of dietary intake

Dietary intake was assessed using the Global Allergy and Asthma European Network (GA2LEN) food frequency questionnaire (FFQ), which was designed as a single, standardized, and internationally validated instrument to ascertain dietary intake facilitating international comparisons [25]. Participants were asked to self-report how often, on average, they had consumed one portion of each of 229 foods and beverages over the last year, selecting one among various response options: rarely or never; once per month; once per week; 2–4 times per week; 5–6 times per week; once per day; and 2+ times per day. The consumption of each item was converted to grams per day to estimate macro- and micro-nutrients and total energy intake (TEI; kcal/day) using the most recent edition of the McCance & Widdowson’s Food Composition Tables [28].

### Definition of the DASH-score

Previous observational, as well as randomized controlled studies have shown the DASH dietary index to be a valid measure to estimate association with various kidney [15, 29], cardiovascular and metabolic outcomes [30]. Moreover, the DASH diet has been recommended by clinical practice guidelines for cardiovascular risk reduction [31]. Adherence to a DASH-style diet was assessed based on eight different food components known for their beneficial or detrimental effects on the risk of hypertension, according to Fung et al. [32]. These food components (Supplementary Table S2), derived from the FFQ and expressed in servings per week, were classified as ‘healthy’ (fruits, vegetables, whole grains, low-fat dairy products, and nuts and legumes) and ‘unhealthy’ (red and processed meats, sodium, and sugar-sweetened beverages (SSB)). The nutrient residual method was used to adjust the DASH components for sex-specific energy intake [33]. The components were then categorised into sex-specific quintiles and ranked from 1 to 5 if belonging to the ‘healthy group’ and from 5 to 1 otherwise. Since the intake of SSB was generally low (interquartile range, IQR: 1-to-7 servings/week) compared to the US, where the score was developed, we used sex-specific tertiles of the distribution to rank this specific component from 3 to 1. The total DASH-score was calculated as the sum of all ranks, ranging from 8 to 36.

### Deriving RRR-scores

We applied RRR to derive dietary patterns informed by a-priori established cardio‑renal‑metabolic pathways. RRR identifies linear combinations of the intake of food groups that explain the maximum variation in a set of selected mediators [18]. Prior to using the RRR method to derive DPs [18], the 229 FFQ items were categorized into 32 groups based on their nutrient composition (Supplementary Table S2) and adjusted for sex-specific TEI using the nutrient residual method [33]. We derived RRR-DPs separately for males and females to capture sex-specific dietary profiles associated with clinical and biochemical markers with known risk for CKD. Selected circulating mediators, chosen for their known association with both diet and kidney health were: glycated haemoglobin (HbA1c), mean arterial pressure (MAP) [34], C-reactive protein (CRP), uric acid, total cholesterol (TC), ferritin, fibrinogen, serum potassium, and haemoglobin (HGB). Ferritin was included as an indicator of long‑term iron storage [35] and because its concentrations are influenced by dietary iron intake [36] and have been reported to show a causal association with eGFRcrea [37]. Hemoglobin was included due to its relationship with dietary iron status [38] and its documented associations with kidney health parameters [39, 40], as well as the proposed role of anemia‑related hypoxia in kidney function decline [41]. Quantile normalization to the last assay was used to account for changes in measurement methods [42]. To prevent confounding, all mediators were age-adjusted and, by means of multilevel models with random intercept for household, we controlled for family-related lifestyle or related environmental effects. The optimal number of RRR derived DPs was identified via screeplot inspection, resulting in 2 DPs. Each DP represented a linear combination of the effects of the food groups on the mediators, summarized through a set of factor loadings. A dietary score (RRR-score) was calculated using the estimated factor loadings as weights for the standardized intake of each food item [43]. RRR modelling and optimal rank estimation were performed using the R package ‘rrpack’ v0.1–12 (https://cran.r-project.org/web/packages/rrpack/index.html).

### Assessment of covariates

Potential confounders were identified via Directed Acyclic Graph (DAG) analysis (Supplementary Figure S1) using the online tool DAGitty (https://www.dagitty.net/dags). We considered: age; sex; BMI (derived from weight and height measured at the study centre); physical activity (continuous Metabolic Equivalent of Task; MET) [44]; smoking habit (Current, Former, Never smoker) [45]; education level (Primary school or no title; Lower secondary school; Vocational school; Upper secondary school; and University or higher); and special diet (Yes, No; indicating whether participants reported following a special diet for medical reasons). To account for non-independency of observations due to relatedness, models were adjusted for the first 10 principal components of the genotyped data with minor allele frequency > 5% [46]. Since menstruation cessation was targeted as a potential effect modifier in the diet-eGFR association, females were classified in two groups based on their menstrual status: Menstruation_Yes_ and Menstruation_No_ according to their response to the question “*Do you still have regular menstrual bleedings?*”. Individuals with missing information on menstrual status (*N* = 32) were assigned Menstruation_No_ if >50 years old and Menstruation_Yes_ otherwise.

### Statistical analyses

To describe and compare the DPs, we estimated the Pearson correlation coefficients between all the DP-scores, including the DASH-score, and between each RRR food group and the scores.

The relationship between the DASH-score, RRR-scores, and eGFR was analysed through linear regression models. To allow direct comparison of the estimates, all dietary scores were z-standardized (mean = 0, variance = 1), allowing associations to be interpreted as per 1 standard deviation (1-SD) change. To reflect differences both in the DPs and disease propensity between males and females, only sex-specific analyses were performed. As informed by the DAG (Supplementary Figure S2), all models were adjusted for age, TEI, physical activity, smoking habits, education, BMI and special diet. To allow for potential non-linear effects of DPs, we additionally fitted generalized additive models (GAMs) with penalized regression splines, with smoothing parameter λ derived via restricted maximum likelihood, using the R package ‘mgcv’ v1.8–40 (https://cran.r-project.org/web/packages/mgcv/index.html). Additionally, considering the potential non-linear association between age, DPs, and the outcome, we incorporated age into the GAMs using a non-linear term.

To investigate the potential influence of menstruation cessation on the relationship between diet and eGFR in females, we performed a preliminary analysis including an interaction term between DASH diet, DP-scores and menstrual status (Menstruation_Yes_; Menstruation_No_). To exclude the possibility of a modification effect of age, an interaction between DP-scores and age was also included in the models. Following the observed evidence that menstrual status was an effect modifier for one of the DP-scores (Supplementary Table S3), all analyses in females were additionally stratified accordingly.

To control for potential residual confounding driven by dietary changes due to any sub-clinical health conditions, we conducted a sensitivity analysis excluding participants (*n* = 283) with sub-clinical diabetes mellitus, reduced kidney function or increased albuminuria (HbA1c > 6.5 mmol/mol or eGFR < 60 mL/min/1.73 m^2^ or urinary albumin-to-creatinine ratio (UACR)>30 mg/g), which we refer to as the Healthy+ sample.

Statistical significance level was set at 0.05. All analyses were performed using the R software v4.1.1 (https://www.r-project.org/).

## Results

### Participant characteristics

Our sample included 3278 females and 2855 males, with similar mean age of 40 years (Table 1). Females had lower median eGFR than males (99.8 versus 104.5 ml/min/1.73 m^2^). Levels of selected mediator biomarkers were generally higher in males, except for CRP and fibrinogen, which were higher in females, and HbA1c and TC, which were equally distributed between sexes. When stratifying females by menstrual status, we observed higher levels of all biomarkers in females with ceased menstruation (Menstruation_No_) except for CRP. As expected, due to higher age (57.2 versus 32.8 years), this group had lower median eGFR (88.4 versus 104.1 ml/min/1.73 m^2^; Table 1).

**Table 1:** Sex-stratified characteristics of the study sample

			Males, *N* = 2855 (46.6%)	Females, *N* = 3278 (53.4%)	Menstruation_Yes_, *N* = 2395 (73.1%)	Menstruation_No_, *N* = 883 (26.9%)
Outcome	eGFR ml/min/1.73 m^2^		104.5 (93.7, 115.1)	99.8 (89.9, 109.4)	104.1 (95.4, 112.7)	88.4 (80.7, 95.8)
Dietary Patterns: scores and components*	DASH score		23 (19, 27)	23 (19, 27)	22 (19, 26)	25 (21, 28)
	Whole grains^		7.5 (4.4, 11.2)	7.5 (4.8, 10.7)	7.1 (4.5, 10.1)	8.7 (5.7, 12.4)
	Nuts and legumes^		1.8 (0.9, 3.2)	1.8 (1, 3.3)	1.9 (1, 3.3)	1.8 (1.1, 3.3)
	Vegetables^		27.9 (19.6, 37.9)	34.9 (26, 45.8)	33.9 (25, 44.9)	38.4 (29.1, 47.9)
	Fruits and Fruit juices^		13.7 (8.1, 22.3)	20.6 (12.4, 33.1)	19.4 (11.7, 30.7)	24.6 (15.4, 39.1)
	Sweetened beverages^		3 (0.8, 7.8)	1.3 (0, 4.8)	1.7 (0.4, 5.2)	0.4 (0, 2.9)
	Red/processed meats^		10 (6.9, 13.7)	6.3 (4.3, 9.1)	6.6 (4.5, 9.4)	5.7 (3.6, 8.5)
	Low-fat dairy^		0.6 (0, 2.8)	0.6 (0, 3.9)	0.7 (0, 3.7)	0.5 (0, 4.2)
	Sodium^		1972.6 (1746.3, 2212.5)	1657.4 (1458.2, 1875)	1670.1 (1465, 1882.5)	1637 (1442.3, 1850.6)
	FDP_1_		—	0.0 (−0.2, 0.2)	0.0 (−0.2, 0.2)	0.1 (−0.1, 0.3)
	FDP_2_		—	0.0 (−0.1, 0.1)	0.0 (−0.1, 0.1)	0.0 (−0.1, 0.1)
	MDP_1_		0.0 (−0.2, 0.2)	—	—	—
	MDP_2_		0.0 (−0.1, 0.1)	—	—	—
	Hba1c, %		5.3 (5.1, 5.5)	5.3 (5.1, 5.5)	5.2 (5.0, 5.4)	5.5 (5.3, 5.6)
	MAP, mmHg		91.0 (85.0, 97.3)	85.3 (79.3, 92.0)	83.7 (78.3, 89.3)	91.3 (83.7, 97.7)
	CRP, mg/dL		0.1 (0.1, 0.2)	0.2 (0.1, 0.3)	0.2 (0.1, 0.3)	0.2 (0.1, 0.3)
	Uric acid, mg/dL		6.0 (5.3, 6.7)	4.4 (3.8, 5.0)	4.3 (3.8, 4.9)	4.7 (4, 5.3)
	TC, mg/dL		203.0 (174.0, 231.0)	203.0 (178.0, 231.0)	194.0 (172.0, 219.0)	231.0 (205.0, 261.0)
	Ferritin, ng/mL		135.7 (84.9, 211.2)	36.1 (18.9, 63.8)	28.8 (15.5, 49.3)	66.8 (42.2, 105.2)
	Fibrinogen, mg/dL		272.0 (240.0, 307.0)	294.0 (262.0, 332.0)	286.0 (255.0, 322.0)	318.0 (288.0, 355.0)
	Potassium, mmol/L		3121.3 (2485.9, 3891.6)	3203.5 (2562.8, 3995.1)	3149.3 (2534.4, 3924.1)	3358.6 (2692.2, 4141.1)
	HGB, g/dL		15.8 (15.2, 16.5)	13.9 (13.3, 14.5)	13.8 (13.2, 14.4)	14.2 (13.5, 14.8)
Quantitative covariates*	Age, years		40.0 (27.5, 51.6)	39.3 (26.4, 51.5)	32.8 (23.2, 42.4)	57.2 (52.9, 63.5)
	Physical activity, MET-min/week		4056.0 (1950.0, 7146.0)	3006.0 (1386.0, 5674.1)	2826.0 (1314.0, 5269.5)	3756.0 (1522.5, 6075.8)
	Total Energy Intake, kcal		1972.2 (1617.9, 2403.1)	1829.8 (1492.0, 2223.9)	1822.7(1480.8, 2215.7)	1855.3(1536.9, 2249.7)
	BMI, kg/m^2^		25.1 (23.1, 27.7)	23.5 (21.3, 26.7)	23.1 (20.9, 26.0)	24.8 (22.3, 28.2)
Categorical covariates**	Smoking habit	*Never smoker*	1512 (53.0)	1897 (57.9)	1400 (58.5)	497 (56.3)
		*Current smoker*	613 (21.5)	626 (19.1)	494 (20.6)	132 (14.9)
		*Past smoker*	730 (25.6)	755 (23.0)	501 (20.9)	254 (28.8)
	Education	*Primary school/no title*	123 (4.3)	184 (5.6)	22 (0.9)	162 (18.3)
		*Lower secondary school*	361 (12.6)	566 (17.3)	341 (14.2)	225 (25.5)
		*Upper secondary school*	626 (21.9)	1042 (31.8)	922 (38.5)	120 (13.6)
		*Vocational school*	1515 (53.1)	1035 (31.6)	696 (29.1)	339 (38.4)
		*University or higher*	230 (8.1)	451 (13.8)	414 (17.3)	37 (4.2)
	Special diet	*Yes*	79 (2.8)	153 (4.7)	113 (4.7)	40 (4.5)
		*No*	2776 (97.2)	3125 (95.3)	2282 (95.3)	843 (95.5)

* Dietary Patterns (scores and components) and quantitative covariates are summarised as median and interquartile range (IQR)

** Categorical covariates are summarised as N (%)

^ Intakes of Dietary Patterns’ components are reported in portions per week

### Description of the DASH score, RRR-scores and their correlations

The overall DASH-score was equally distributed among sexes by design, as it was derived from sex-specific food intake quintiles. With respect to the DASH-score relevant dietary components, we observed a higher intake of ‘healthy’ food items (nuts and legumes, vegetables, fruit and fruit juices) in females, while males reported a higher intake of ‘unhealthy’ items, especially sodium, and red and processed meat (Table 1). Stratification of females by menstrual status revealed relevant differences in dietary habits, with lower intake of sodium, red and processed meat, and SSB after menstruation cessation, combined with higher intake of refined grains, vegetables, fruits and fruit juices.

**Fig. 1:**
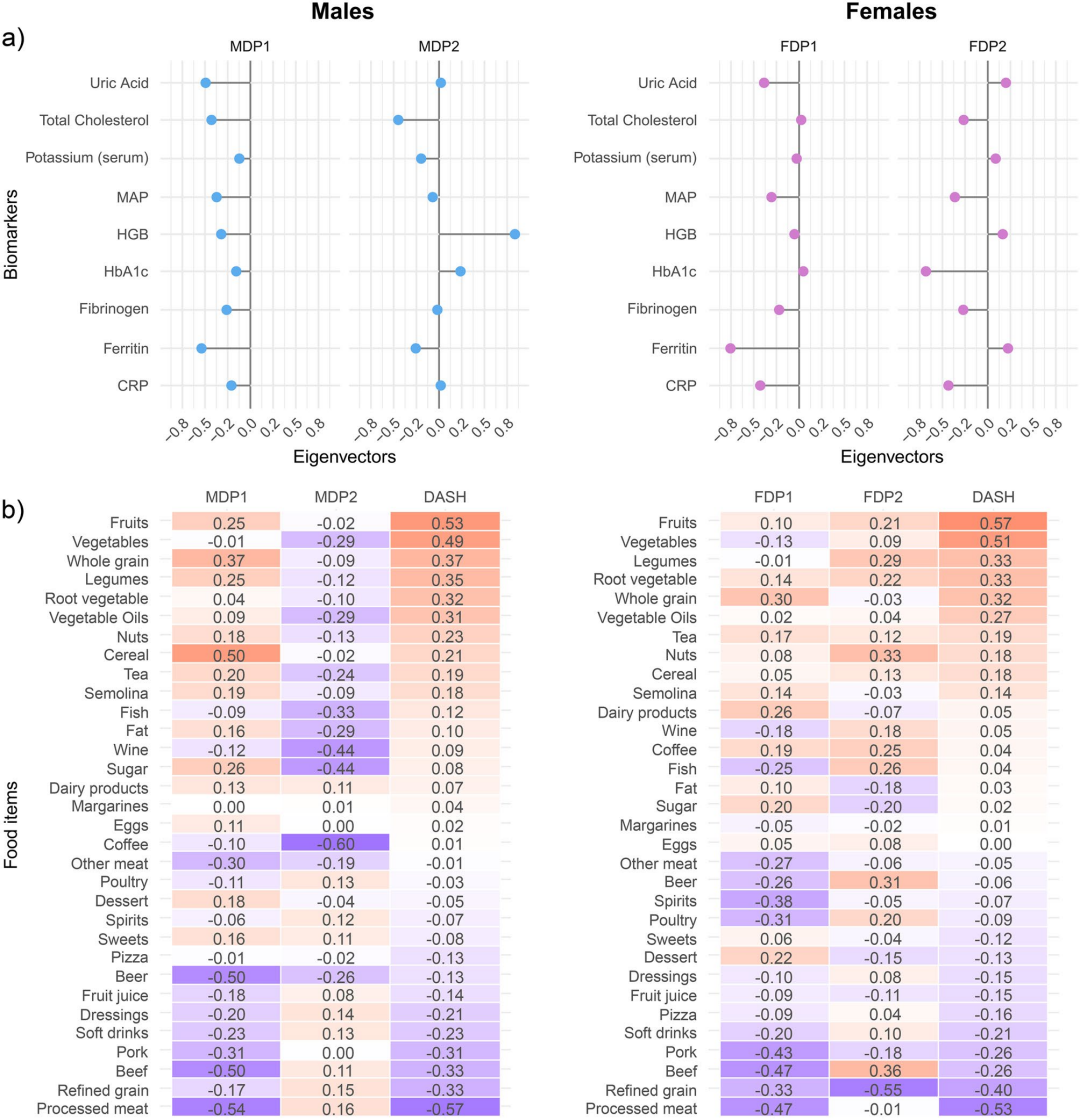
Representation of the dietary patterns (DPs). **a**) RRR-based eigenvectors summarizing the influence of the biomarkers in defining the respective DP-scores in males and females*. **b**) Heatmaps of the Pearson’s correlation coefficients between RRR food groups and DP scores. Footnote: *the explained variance in biomarkers by the two dietary patterns was 67.3% in males and 60.9% in females

A general description of all dietary components used to derive RRR-scores is provided in Supplementary Table S4. We identified two RRR-scores for males and two different ones for females, which we labeled as MDP_1_ and MDP_2_ (explaining 67.3% of the biomarkers variance) and FDP_1_ and FDP_2_ (explaining 60.9% of the biomarkers variance), respectively (Fig. 1). A high MDP_1_ reflected lower levels of all biomarkers. It was driven by higher intake of whole grains and cereals, sugar, fruits, and legumes, and lower intake of beer, and red and processed meat. High MDP_2_, which was associated with higher levels of HGB and HbA1c, and lower levels of TC, serum potassium, and ferritin, was mainly driven by low intake of coffee, alcohol (wine and beer), fish, fat, vegetable-based oils, and vegetables. In females, high FDP_1_ was associated with lower levels of uric acid, MAP, fibrinogen, ferritin, and CRP. FDP_1_ was driven by higher intake of whole grains, and dairy products, and by low intake of red and processed meat, refined grains, fish, poultry and alcohol (beer and spirits). High FDP_2_, which corresponded to higher levels of uric acid, HGB, and ferritin levels, and low levels of TC, MAP, HbA1c, fibrinogen and CRP, was driven by higher intake of beef, nuts, beer, legumes, fish, and coffee, and lower intake of refined grain.

While the correlations between RRR-scores were null by design, in males, we observed moderate positive correlations of the DASH-score with MDP_1_ (r_DASH-MDP1_ = 0.52) and a weak negative correlation with MDP_2_ (r_DASH-MDP2_ = −0.27). In females, we observed a moderate positive correlation of the DASH-score with FDP_1_ (r_DASH-FDP1_ = 0.34) and a weak positive correlation with FDP_2_ (r_DASH-FDP2_ = 0.18).

### Associations between DASH-score, RRR-scores and eGFR

The preliminary analysis in females (Supplementary Table S3) investigating potential effect modification by age and menstrual status showed a significant interaction between menstrual status and FDP_1_ (${\rm{\hat \beta }}$: 2.12 ml/min/1.73 m^2^; 95%CI: 0.85, 3.40).

**Fig. 2:**
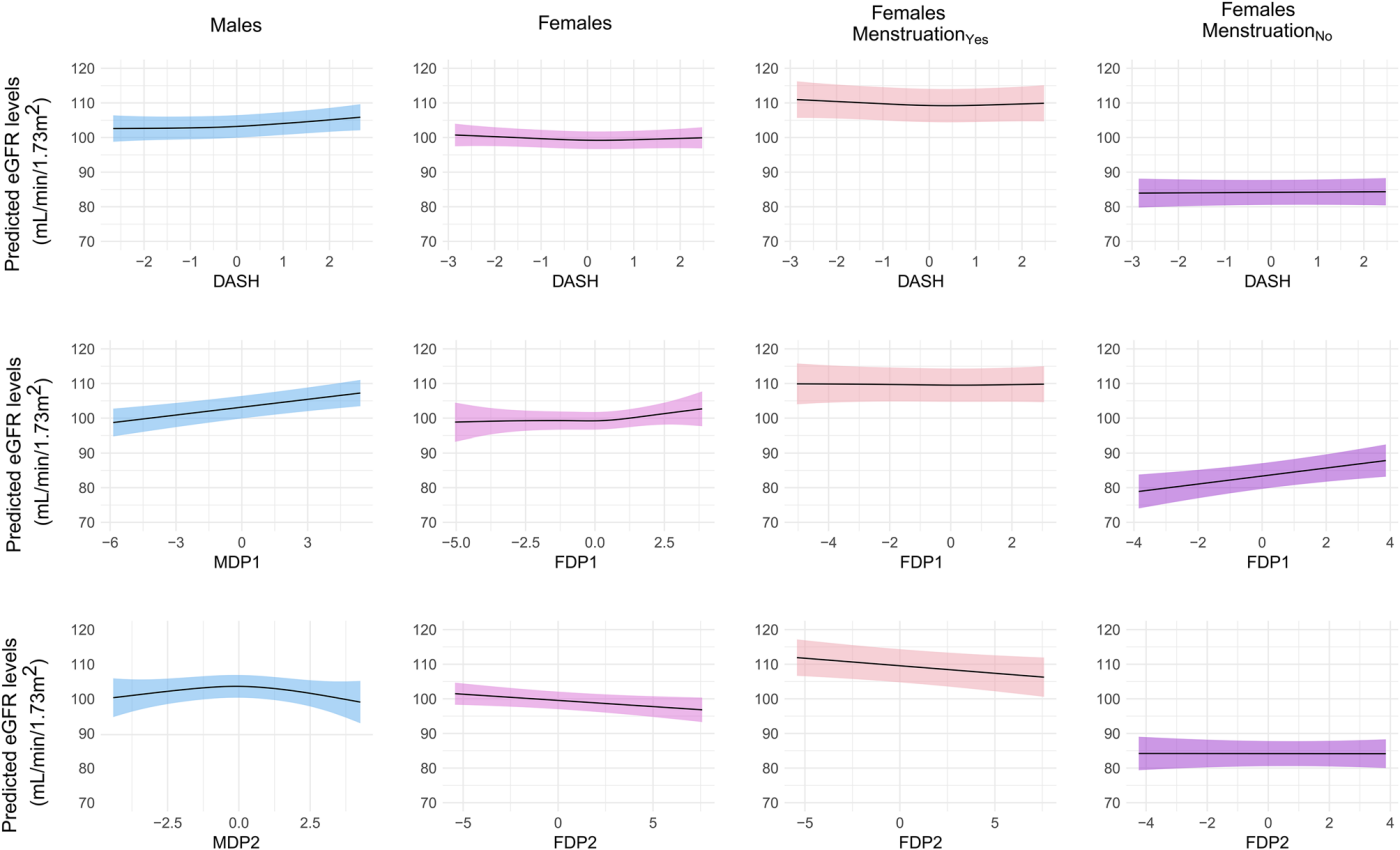
Plots of predicted eGFR levels by the different scores, as obtained from the fitted GAMs

Final associations between DPs and eGFR are summarized in Table 2. In males, each 1-SD increase in the DASH-score was associated with 0.64 ml/min/1.73 m^2^ higher eGFR (95%CI: 0.22, 1.06). The GAM’s estimated degrees of freedom (edf) were 1.8, suggesting a slightly quadratic association (Fig. 2). In females, no association was found between the DASH-score and eGFR, independent of menstrual status.

**Table 2:** Results of the linear (LM) and generalized additive models (GAM)

			LM		GAM	
			Effect (95% CI)	p-value	edf*	p-value
Males		DASH	0.64 (0.22, 1.06)	0.0029	1.8	0.0082
		MDP_1_	0.75 (0.38, 1.13)	0.0001	1.0	0.0001
		MDP_2_	0.04 (−0.42, 0.50)	0.8676	2.4	0.1149
Females		DASH	−0.18 (−0.54, 0.19)	0.3448	2.01	0.2450
		FDP_1_	0.24 (−0.16, 0.64)	0.2345	2.46	0.2319
		FDP_2_	−0.36 (−0.7, −0.02)	0.0392	1.00	0.0386
Menstrual status	Menstruation_Yes_	DASH	−0.26 (−0.69, 0.17)	0.2389	1.9	0.2366
		FDP_1_	−0.01 (−0.48, 0.46)	0.9593	1.3	0.9151
		FDP_2_	−0.43 (−0.83, −0.04)	0.0326	1.0	0.0327
	Menstruation_No_	DASH	0.06 (−0.63, 0.76)	0.8610	1.0	0.8358
		FDP_1_	1.11 (0.34, 1.88)	0.0049	1.0	0.0035
		FDP_2_	0.00 (−0.65, 0.65)	0.9969	1.0	0.9801

LM: effects of DPs on eGFR in ml/min/1.73 m^2^ with 95% confidence intervals (CI) and *p*-values; GAM: estimated degrees of freedom (edf) and p-values

Symbols and abbreviations: CI, confidence interval of the effect; edf, estimated degrees of freedom

Regarding the RRR-scores in males, MDP_1_ was positively associated with eGFR (${\rm{\hat \beta }}$ = 0.75 ml/min/1.73 m^2^; 95%CI: 0.38, 1.13); no association was observed between MDP_2_ and eGFR. In females, we observed negative associations between FDP_2_ and eGFR, both overall (${\rm{\hat \beta }}$: −0.36 ml/min/1.73 m^2^; 95%CI: −0.70, −0.02), and in the Menstruation_Yes_ group (${\rm{\hat \beta }}$: −0.43 ml/min/1.73 m^2^; 95%CI: −0.83, −0.04). In the Menstruation_No_ group, results showed a positive association between FDP_1_ and eGFR (${\rm{\hat \beta }}$: 1.11 ml/min/1.73 m^2^; 95%CI: 0.34, 1.88). The GAMs confirmed both significance and linearity of the results (edf = 1) (Fig. 2).

Findings of our sensitivity analyses for the Healthy+ subgroup did not differ substantially from the overall sample, showing similar sample characteristics (Supplementary Table S5), as well as results between DPs and eGFR (Supplementary Table S6). In males, we confirmed the positive association with eGFR for the DASH-score (${\rm{\hat \beta }}$: 0.67 ml/min/1.73 m^2^; 95%CI: 0.24, 1.09) and MDP_1_ (${\rm{\hat \beta }}$: 0.83 ml/min/1.73 m^2^; 95%CI: 0.44, 1.21), and the null association between MDP_2_ and eGFR. In females, FDP_2_ was negatively associated with eGFR both overall (${\rm{\hat \beta }}$: −0.39 ml/min/1.73 m^2^; 95%CI: −0.73, −0.04), and in the Menstruation_Yes_ group (${\rm{\hat \beta }}$: −0.46 ml/min/1.73 m^2^; 95%CI: −0.87, −0.05). In the Menstruation_No_ group, FDP_1_ appeared to be linearly associated with eGFR in the GAM, however this association was not confirmed by the linear model. In all models we observed a slight increase in the magnitude of the significant associations. GAMs confirmed both linearity and significance of those associations.

## Discussion

In this investigation conducted among a generally healthy population, we found sex and menstrual status to play a significant role in CKD prevention through diet. In males, adhering to the DASH-style diet, as well as to a diet characterized by higher intake of plant-based foods and lower intake of alcohol, red and processed meat was associated with higher eGFR, reflecting better kidney function. In females, the associations between the DPs and eGFR varied by menstrual status, except for the DASH-style diet, which did not show any association with eGFR.

The DASH diet has been favourably linked to relevant risk factors associated with CKD onset, such as insulin resistance, hypertension, and dyslipidemia, and might therefore also be protective against kidney dysfunction [14, 32]. A recent meta-analysis of six observational studies estimated that higher adherence to the DASH-score was associated with a 23% lower risk of developing CKD [15]. However, this association was more pronounces in studies where the index was extracted from nutrients rather than food groups. We found that the food group-based DASH-score was associated with higher eGFR in males only. Although the DASH score was similarly distributed among females and males, the differences observed for the actual consumption of the DASH components between sexes, specifically the higher consumption of ‘healthy’ items in females and ‘unhealthy’ items in males, might partially explain the null findings observed in females.

To the best of our knowledge, this is the first investigation using cardio-renal biomarkers to identify disease-oriented DPs using RRR in a generally healthy population. In a population-based cohort Cai and colleagues [21] derived RRR-based DPs directly related to eGFR at baseline and evaluated its association with eGFR decline and CKD incidence at five-year follow-up. Among females, they observed a DP characterized by high consumption of eggs, cheese, and legumes, and low consumption of sweets, white meat, and commercially prepared dishes. In males, the DP was characterized by high consumption of cheese, bread, milk, fruits, vegetables, and beer and low consumption of white and red meat. Higher adherence to the scores was associated with lower risk of eGFR decline in both sexes, which aligns with our results. Kurniawan et al. [22] estimated kidney function-oriented DPs including waist-to-hip ratio, triglycerides, LDL-C, TC/HDL-C, calcium, phosphorus, creatinine, and blood urea nitrogen as mediators, and identified a corresponding DP associated with increased risk of impaired kidney function among older individuals with CKD. High adherence to this DP was characterized by higher intake of preserved vegetables, processed meat or fish, rice or flour products, meat, soy sauce, organ meats, fried rice or flour products, and instant noodles, along with lower intakes of fruits, dark-coloured vegetables, bread, and beans or bean products. Although we used a different set of biomarkers and conducted the analyses among a population free of CKD and related comorbidities, our results partially align with their findings, specifically in males.

Overall, in both sexes any kind of meat and fish, as well as whole grains, fruits, vegetables, legumes, and nuts, played a strong role in the RRR-based DP composition. Animal-sourced proteins, processed foods, and refined grains are known to result in high acidic loads when metabolized [22], in contrast with vegetable-sourced proteins, which lead lower acidic loads [47]. High dietary acidic load may consequently induce kidney adaptive mechanisms to increase acid excretion, thereby promoting renal injury [16]. Moreover, a higher plant-to-animal protein ratio in the diet may protect against kidney dysfunction by increasing serum bicarbonate levels and reducing fibroblast growth factor 23 levels [14, 48]. We have observed a similar impact of poultry and fish consumption in two of our previous analyses investigating associations between nutrient-related DPs and kidney function and damage [49], as well as the impact of different protein sources on eGFR [50]. Although regular fish intake is suggested to promote general and specifically cardiovascular health, some uncertainties remain regarding its health benefits, which could be related to the type and preparation of the fish and whether it is processed [51]. Additionally, plant-based food may exert beneficial effects on kidney function through higher intake of fiber, magnesium, phosphorus and potassium, which have an impact on inflammatory cytokines [15], endothelial disfunction [52], and blood pressure levels [53], all factors contributing to impaired kidney function.

In females, we found potential evidence of the diet-eGFR association to be modified by menstrual status. While FDP_1_ was associated with higher levels of eGFR in the Menstruation_No_ group only, FDP_2_ was associated with lower levels of eGFR in the Menstruation_Yes_ group only. Higher adherence to FDP_1_ corresponded to lower levels of uric acid, MAP, fibrinogen, ferritin, and CRP. In contrast, higher adherence to FDP_2_ corresponded to higher levels of uric acid, HGB, and ferritin levels, and low levels of TC, MAP, HbA1c, fibrinogen and CRP. These observed differences might suggest a role of iron metabolism and ferritin levels, among others, that could modify the relationship between diet and kidney function in females. A recent Japanese study revealed that both low and high serum ferritin levels are linked to increased risk of adverse kidney outcomes compared to intermediate levels [54]. Elevated serum ferritin levels, in particular, may pose a risk of iron overload [55, 56]. Moreover, ferritin is closely related to other inflammation markers [57]. Together with ferritin, in fact, CRP and fibrinogen were also relevant for FDP_1_ profile, supporting evidence from previous studies suggesting a potential role of inflammation on kidney function levels [58]. In addition, the concurrent presence of high serum uric acid and CRP levels could be associated with an increased risk of type II diabetes [59]. Therefore it seems reasonable to observe higher eGFR levels in individuals with a dietary profile (FDP_1_) associated with lower levels of these markers. Nevertheless, it should be considered that baseline levels of biomarkers differed by menstrual status. Females with ceased menstruation exhibited higher levels of all biomarkers, except for CRP.

The comparison of the DPs revealed a positive correlation between the RRR-based MDP_1_ and the DASH score, suggesting that our population-specific, disease-oriented DPs in males, reflect the dietary guidelines defined to contrast hypertension (DASH). Overall, the RRR-scores were more strongly associated with eGFR than the DASH-score. The flexibility of the approach, allowing to shape DPs in a disease-related manner, potentially could lead to the detection of unknown associations and complex mechanisms. For example, FDP_2_ was weakly correlated with the DASH-score, but it highlighted a relevant pattern related to lower eGFR in the Menstruation_Yes_ group. Recent studies highlighted sex differences in disease biomarkers, as observed in CVDs [60]. Future studies should therefore reconsider the appropriateness of selecting common biomarkers for males and females. This further emphasizes the recent recognition of sex differences in the aetiology, mechanisms, epidemiology, and treatment of CKD [8]. Moreover, females are less likely to receive diagnoses, monitoring, and management compared to males [6]. While evidence suggests that sex influences various aspects of treatment and complications in later-stage kidney disease, research on its impact on earlier CKD stages is limited [61]. While CKD is more prevalent in females [26], kidney function declines more rapidly in males [62]. It has been hypothesized that these sex differences may be attributed to the protective effects of endogenous estrogens against the deleterious effects of testosterone on kidney function and structure, as well as the generally healthier lifestyles observed in females [8]. In addition, the role of menstrual status, probably mediated by ferritin and inflammation markers, underscores the need for further investigation concerning CKD in females.

Strengths of our investigation are the large sample size and the use of a validated FFQ. Detailed information on medical history and lifestyle allowed us to control for an extensive range of relevant confounders. Finally, we included and compared two approaches to evaluate DPs in our sample. This enabled us to obtain a complementary and comprehensive overview of the role of diet in the association with eGFR. Particularly, the DASH index has been shown to be a valid measure to estimate associations with various health outcomes, including kidney and cardiovascular disease [15, 29–31]. Therefore, while using the DASH index simplified the interpretation and direct comparison of our findings with other studies, the complementary usage of a hybrid approach as RRR allowed as to derive our DPs in a more flexible and exploratory way. Specifically, it allowed us to consider the multifactorial and complex nature of CKD, often characterized by multiple comorbidities, which could share aspects of their pathophysiology and require the formulation of comprehensive dietary recommendations.

The main limitation of our investigation is the cross-sectional nature of the data. The one-time assessment of self-reported dietary intake of participants might be affected by seasonality or recall bias. Moreover, the simultaneous collection of health information and dietary intake could prevent any causal interpretations of our findings. We therefore focused our analyses on individuals who self-reported of being free from CKD, hypertension and diabetes. Along with adjustments made for special diet, this should have mitigated the risk of reverse causation. Furthermore, results were robust to exclusion of individuals with subclinical diabetes mellitus, reduced kidney function and increased albuminuria, confirming that CKD, given its silent nature, especially in the early stages, is unlikely to stimulate dietary changes prior to formal diagnosis. However, the choice to focus on healthy individuals might have increased the outcome’s measurement error. The difference between estimated and measured GFR is usually more pronounced in healthy individuals than in those with impaired kidney function [63]. In balancing the two competing risks of collider bias, which could cause reverse causation if individuals with diabetes, hypertension or CKD are included, and reduced clinical relevance due to an increased measurement error, it is worth noting that creatinine-based estimates of GFR remain relevant for healthy individuals in the general population. For instance, genomic studies of eGFR in the general population, which were enriched of individuals with eGFR > 90 mL/min/1.73 m^2^, were characterized by an outstanding enrichment of genes expressed in kidney tissues compared to other tissues [64]. In fact, even in healthy individuals with non-critical eGFR levels, modest eGFR reductions can predict a higher occurrence of adverse outcomes [65], which supports overall validity of our analyses. Furthermore, at high GFR levels, the measurement error due to GFR estimation does not seem to exhibit systematic over- or underestimation [66]. Therefore, our results should be biased toward the null, preventing the identification of additional significant findings, which could potentially emerge with larger sample sizes, but not causing the identification of false positive results. Another limitation related in the absence of measured GFR and cystatin C in our study is the use of serum creatinine, which is influenced by muscle mass and protein intake. However, the use of 12-hour fasting plasma samples, as done by the CHRIS study, has been shown to mitigate the acute effect of meat consumption on serum creatinine levels [67]. The last limitation related to the common challenge of extrapolating sodium intakes from FFQs [68]. This could have led to an underestimation of detrimental effects of lower adherence to the DASH diet, as well as other processed food items, rich in sodium and other additives [69]. Furthermore, our analysis included some missing data. Individuals with a substantial proportion of missing values were excluded, and the remaining missing data were handled using multiple imputation by chained equations, which assumes missingness at random. If this assumption is violated, some degree of bias may persist. However, given the very small proportion of missing data, any resulting impact on the estimates is expected to be minimal. Moreover, as in any observational study, although we tried to carefully control for the role of potential confounders, the possibility of residual confounding from unmeasured variables cannot be ruled out. Finally, our analysis was conducted in a population sample from a small, Alpine geographical area. Given the strong relation between diet and culture, it is unknown to which extent our findings are generalizable to other geographical and cultural contexts as well as to non-European populations. Finally, extension of our investigation to a longitudinal context would help to better clarify the possible causal role of dietary patterns of kidney function. While the CHRIS study is achieving a 10-year follow-up, which will enable such an investigation [24], a similar analysis from our group on incident CKD cases has partially confirmed the relevance of similar DPs for kidney health [70].

## Conclusion

A DASH-style diet and a disease-oriented dietary pattern characterized by higher intake of plant-based foods, and lower intake of beer, SSBs, red and processed meat, were associated with higher eGFR in males without CKD, diabetes and hypertension. In females, associations between RRR-based DPs and eGFR were modified by menstrual status. Overall, results highlight the need to consider sex-differences when studying the relationship between diet and kidney health.

## Electronic supplementary material

Below is the link to the electronic supplementary material.

Supplementary Material 1

## Data Availability

Data and samples can be requested for clearly defined research via the CHRIS Portal ([https://chrisportal.eurac.edu](https://chrisportal.eurac.edu)). For questions, contact [rebecca.lundin@eurac.edu](mailto:rebecca.lundin@eurac.edu) .
